# CD24 enrichment protects while its loss increases susceptibility of juvenile chondrocytes towards inflammation

**DOI:** 10.1186/s13075-016-1183-y

**Published:** 2016-12-12

**Authors:** Jieun Lee, Piera Smeriglio, Jason Dragoo, William J. Maloney, Nidhi Bhutani

**Affiliations:** Department of Orthopaedic Surgery, Stanford University School of Medicine, Stanford, CA 94305 USA

**Keywords:** Cartilage, CD24, inflammation, OA

## Abstract

**Background:**

Diseases associated with human cartilage, including rheumatoid arthritis (RA) and osteoarthritis (OA) have manifested age, mechanical stresses and inflammation as the leading risk factors. Although inflammatory processes are known to be upregulated upon aging, we sought to gain a molecular understanding of how aging affects the tissue-specific response to inflammation. In this report, we explored the role of cluster of differentiation 24 (CD24) in regulating differential inflammatory responses in juvenile and adult human chondrocytes.

**Methods:**

Differential cell-surface CD24 expression was assessed in juvenile and adult chondrocytes along with human induced pluripotent stem cell (hiPSC)-derived neonatal chondrocytes through gene expression and fluorescence-activated cell sorting (FACS) analyses. Loss of function of CD24 was achieved through silencing in chondrocytes and the effects on the response to inflammatory cues were assessed through gene expression and NFκB activity.

**Results:**

CD24 expression in chondrocytes caused a differential response to cytokine-induced inflammation, with the CD24^high^ juvenile chondrocytes being resistant to IL-1ß treatment as compared to CD24^low^ adult chondrocytes. CD24 protects from inflammatory response by reducing NFκB activation, as an acute loss of CD24 via silencing led to an increase in NFκB activation. Moreover, the loss of CD24 in chondrocytes subsequently increased inflammatory and catabolic gene expression both in the absence and presence of IL-1ß.

**Conclusions:**

We have identified CD24 as a novel regulator of inflammatory response in cartilage that is altered during development and aging and could potentially be therapeutic in RA and OA.

**Electronic supplementary material:**

The online version of this article (doi:10.1186/s13075-016-1183-y) contains supplementary material, which is available to authorized users.

## Background

Cartilage degenerative diseases like rheumatoid arthritis (RA) and osteoarthritis (OA) have inflammation, age and obesity as causal risk factors; however, the precise molecular mechanisms underlying these risk factors are ill-understood. No disease-modifying drug is available for OA, while the drugs available for RA are not equally effective in all patients. Increased molecular understanding of the causal factors will therefore be beneficial in both diseases.

Very few studies have systematically evaluated the age-dependent changes in human tissue including cartilage due to limited availability of human cartilage tissue. These studies are particularly pertinent to cartilage because cartilage regeneration is inefficient even in healthy young adults, often leading to OA, even though pediatric patients demonstrate superior cartilage repair. Recently, allogeneic juvenile cartilage (from donors below 13 years of age) has even been successfully utilized for repair of focal cartilage defects. Upon phenotypic and functional characterization of juvenile and adult chondrocytes, it was found that juvenile chondrocytes demonstrate increased cell proliferation and extracellular matrix (ECM) generation as compared to the adult chondrocytes [1]. The molecular factors responsible for these functional differences that define the regenerative capacity of juvenile and adult chondrocytes have, however, not been characterized.

Another key question that remains unanswered is how age-related changes modulate the cell and tissue-specific response to inflammation. Inflammaging, i.e. systemic upregulation of inflammatory cues with aging is a well-documented phenomenon. For example, plasma levels of the pro-inflammatory cytokine, interleukin-6 (IL-6) are low in young adults and begin to increase in healthy people at about 50–60 years of age [2]. Inflammaging is associated with many forms of age-related pathological conditions, such as neurodegeneration, atherosclerosis, metabolic syndrome, diabetes mellitus and conditions affecting the musculoskeletal system (i.e. osteoporosis, OA and RA) [3, 4]. However, it remains unclear whether the age-related changes in tissues render them increasingly susceptible to the inflammaging cues, thereby leading to increased inflammation-mediated damage in aging tissues.

To address the effects of age-related changes in cartilage regeneration and degeneration, we have recently performed genome-wide gene expression profiling of juvenile and adult chondrocytes [5]. Striking phenotypic and functional differences have been reported between juvenile and adult human chondrocytes, demonstrating the characteristic functional differences such as increased ECM production by the juvenile chondrocytes compared to adult chondrocytes [6, 7]. In order to dissect the underlying biological differences that lead to the observed increased regenerative capability of juvenile chondrocytes [5, 7], we have compared the molecular differences between the juvenile and adult articular chondrocytes by utilizing exon microarrays to determine their global gene expression profiles [5].

Multiple previous studies have identified cell-surface cluster of differentiation (CD) molecules including CD44 (the hyaluron receptor), CD90 (Thy 1) and CD49 (alpha integrins) to play critical roles in and be markers of the chondrogenic capacity of chondrocytes [8–11]. Among approximately 600 factors that were differentially upregulated in juvenile chondrocytes, our studies identified CD24 to be highly enriched in juvenile chondrocytes with expression being lost with age such that the adult chondrocytes only demonstrate a modest expression. CD24 is a small, heavily glycosylated and glycosyl-phosphatidylinositol (GPI)-anchored cell-surface protein that is a co-stimulator for antigen-specific T cell responses and a differentiation marker for B cells [12, 13]. Importantly, polymorphisms of human CD24 are associated with risk and progression of several autoimmune diseases, multiple sclerosis and RA [14–17]. In this study, we provide evidence for a novel role for CD24 in cartilage function whereby it can negatively modulate NFκB activity and hence the response to inflammatory cues.

## Methods

### Chondrocyte isolation and culture

Four individual juvenile and four individual adult samples were analyzed in the study. The juvenile articular chondrocytes (from a 24-week old fetus (designated as J1), a 6-year-old child (J2), and a 6-month-old (J3) and an 18-month (J4) infant) were purchased from Lonza (Clonetics™, Lonza Walkersville, Inc.). These primary chondrocytes (four biological samples) were then thawed, expanded for 5–7 days as high-density monolayers and utilized for experiments.

Adult articular chondrocytes were harvested from grossly normal pieces of cartilage discarded during notchplasty or debridement from patients with no prior history of OA under protocols approved by the human subjects Institutional Review Board of Stanford University. The four adult samples utilized in the study were from a female 27-year-old (A1), a male 35-year-old (A2), a male 18-year-old (A3), and a female 39-year-old (A4) individual. The cartilage pieces were dissected and the chondrocytes were dissociated from the matrix as described previously [18]. All chondrocytes were cultured in high-density monolayers for limited passages (under four passages), as described previously [5, 7].

Five different batches of human induced pluripotent stem cell-derived chondrocytes (hiChondrocytes) were derived by differentiation of human induced pluripotent stem cells (hiPSC) as previously described and characterized [10]. Five different batches were utilized to ensure reproducibility. The hiChondrocytes were cultured for up to four passages and used to test for CD24 expression.

### Flow cytometry

Cells were dissociated to a single-cell suspension using TrypLE (Life Technologies) and fixed in BD Cytofix buffer (BD Biosciences) for 20 minutes at room temperature. For permeabilization, cells were washed and incubated with BD Permeabilization/Wash buffer (BD Biosciences) at 1 × 10^7^ cells per 1 mL for 10 minutes. Cells were stained by incubating with anti-human CD24-PE (BD Biosciences) for 30 minutes. The antibody was diluted according to manufacturer’s instruction. Stained cells were scanned using an LSRII flow cytometer and analyzed with FlowJo software.

### Quantitative real-time PCR

RNA was isolated with the RNeasy kit. First-strand cDNA was primed with oligo (dT) primers and qPCR was performed with Taqman primer sets from Applied Biosystems (Foster City, CA). Relative expression levels were normalized to glyceraldehyde-3-phosphate dehydrogenase (GAPDH) and ribosomal 18S RNA. Then expression levels were calculated using the method 2 − Δ cycle threshold (Ct) [19].

### Immunohistochemical analysis

Adult chondrocytes tissue were fixed in 4% paraformaldehyde (Sigma) then mounted with paraffin. Tissue sections were de-paraffinized with 100%, 90% and 70% ethanol and permeabilized in cold methanol (Sigma). After blocking in PBS containing 1% BSA, tissue sections were incubated overnight with primary antibody (anti-CD24, 1:100, BD). The following day cells were washed in PBS and incubated for 1 hour in secondary antibody (Alexa 594 goat anti-rabbit 1:250, Invitrogen) and cellular DNA was counterstained with 4',6-diamidino-2-phenylindole (DAPI) (Life Technologies).

### IL-1ß treatment

Chondrocytes were plated at 5 × 10^5^ cells per well in duplicates in 6-well plates. After 24 hours, cells were treated with control or IL-1ß (10 ng/mL) in complete media for 2 days.

### ShCD24 lentiviral preparation and infection

HEK293FT cells were plated at a density of 6 × 10^6^ cells per T225 flask and incubated overnight. Cells were transfected with 7.5 μg of VSV-G, 5.7 μg of TAT, 7.5 μg of Rev, 30 μg of Gag/Pol and 15 μg of shCD24 lentiviral plasmid (Sigma) with Lipofectamine. The supernatant was collected 48 hours after transfection and filtered through a 0.45-μm filter. Following spinning at 17,100 rpm for 2 hours 20 minutes, the viral pellet was resuspended to make × 100 stock solutions. To knock down CD24 expression, chondrocytes were seeded at 5 × 10^4^ cells per well of a 6-well dish a day before transduction. The medium was replaced with 100 multiplicity of infection (MOI) virus-containing supernatant supplemented with 8 μg/mL polybrene, and incubated for 24 hours. The transduced chondrocytes were then cultured in chondrocyte medium and we evaluated the reduction in CD24 using real-time quantitative PCR.

### NFκB luciferase assay

Chondrocytes (3 × 10^5^/well) were subjected to either shNTC or shCD24 infection with or without IL-1ß (10 ng/mL). Cells were transfected with pNFκB-Luc (Agilent Technologies, Santa Clara, CA, USA) and pFC-MEKK as a positive control plasmid by using Fugene 6 (Promega, Madison, WI, USA) and after 24 hours, cells were assayed using the Bright-Glo Luciferase Assay System (Promega) with a luminometer. In order to account for any differences in transfection efficiency, the PathDetect reporting system (Agilent Technologies, Santa Clara, C, USA) utilized an unrelated reporter pFC-MEKK containing a MEKK kinase from the constitutive cytomegalovirus (CMV) promoter responsible for activating luciferase transcription as a positive control. We used 1ug/uL of pCIS-CK negative control plasmid containing the luciferase reporter gene and no cis-acting DNA elements as a negative control. Cells with or without CD24 (i.e. non-target and shCD24-infected cells, respectively) were transfected with either 1 ug/uL of pNFκB-Luc (experimental) or 1 ug/uL of pFC-MEKK, i.e the positive control. The luciferase activity for the positive control was utilized to normalize the pNFκB-Luc activity.

### Statistical analyses

Data are reported as the mean ± standard error of the mean (SEM) and followed the normal distribution of independent experiments, which were done at least three times. Statistical analysis was performed using the two-tailed Student’s *t* test and one-way analysis of variance (ANOVA) followed by the Bonferroni’s test for multiple-comparisons. *P* values less than 0.01 were considered significant (details in Additional file 1).

## Results

### CD24 expression is high in juvenile chondrocytes compared to adult chondrocytes

The juvenile and adult samples used in the previous and present study were characterized in detail for chondrogenic gene expression (high Sox9 and Col2a1 expression), lack of fibrocartilage or dedifferentiation markers (no increase in Col1a or Col10a1 expression) and the characteristic functional differences between the adult and juvenile chondrocytes, such as higher proliferation and ECM production as described previously [7].

**Fig. 1:**
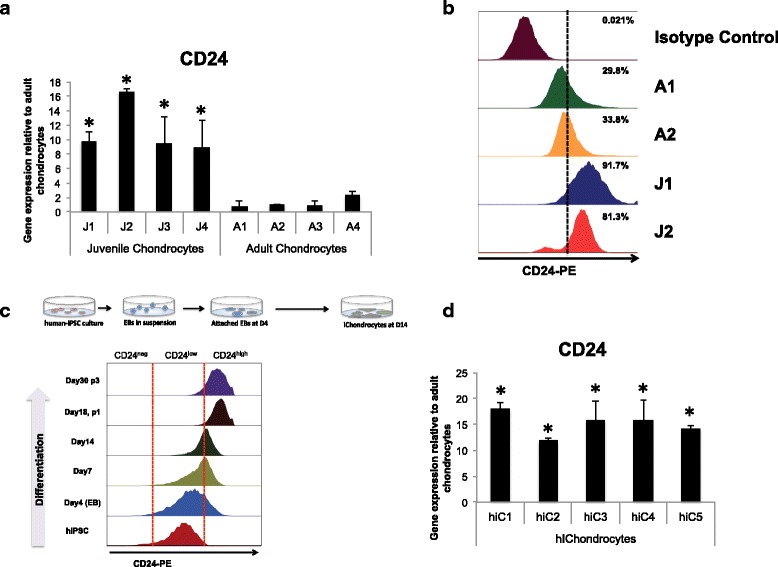
**a** Gene expression for CD24 is higher in juvenile chondrocytes (*J1*–*J4*: donor ages 24 weeks, 6 years, 6 months and 18 months) as compared to adult chondrocytes (*A1* − *A4*; donor age 18 (male), 35 (male), 27 (female) and 39 (female) years); *p < 0.01. **b** Flow cytometry analyses (*right*) show a higher percentage of CD24-positive cells in juvenile chondrocyte populations (*J1*, *J2*), 92% and 81%, respectively, as compared to 30% and 34% in adult chondrocytes (*A1*, *A2*). **c** CD24 expression is gradually increased during chondrocyte differentiation from human induced pluripotent stem cells (*iPSCs*). **d** Human induced pluripotent stem cell-derived chondrocytes (*hiChondrocytes*) (*hiC1*–*hiC5*) expresses CD24 to a greater extent than adult chondrocytes. Gene expression is relative to adult articular chondrocytes (AC)1 in the absence of IL-1ß for each respective gene; *p < 0.01

Among the identified factors, the cell-surface receptor CD24 showed 8-fold to 10-fold increased expression in juvenile chondrocytes as compared to the adult chondrocytes. To validate the differential enrichment of CD24 in juvenile chondrocytes, we examined CD24 expression at a transcript level by quantitative PCR and at a single cell protein level utilizing FACS analyses (Fig. 1a). Gene expression analyses on juvenile and adult articular chondrocytes from four different donors each (see “Methods”) confirmed an 8-fold to 10-fold increase in CD24 expression in the juvenile chondrocytes compared to the adult chondrocytes (Fig. 1a).

Juvenile (*n* = 2 donors) and adult chondrocytes (*n* = 2 donors) were further utilized for the single cell flow cytometry analyses. FACS analyses demonstrated that the juvenile chondrocytes consisted of a uniformly CD24^high^ population (81–92%). The majority of the adult chondrocytes on the other hand showed a lower level of CD24 expression with a smaller population (30 − 34%) being CD24^high^ (Fig. 1b). Supporting these observations in isolated chondrocytes, immunostaining for CD24 in dissected adult cartilage slices showed CD24 expression but not in all the chondrocytes (Figure S1, see Additional file 2). Collectively, these data confirmed that CD24 is a cell surface receptor enriched in juvenile chondrocytes as compared to adult chondrocytes.

As CD24 appeared to mark juvenile chondrocytes, we next assessed CD24 expression in the hiPSC-derived chondrocytes (hiChondrocytes). We have previously established methods to differentiate hiPSC into articular-like chondrocytes (hiChondrocytes) and characterized the chondrogenic phenotype of the hiChondrocytes in terms of gene and protein expression and their ability to engineer cartilage in vitro and in vivo [10]. We therefore hypothesized that these hiChondrocytes will mimic developmentally younger chondrocytes and may have enriched levels of CD24. To test this hypothesis, we investigated CD24 expression during differentiation of hiPSC to chondrocytes in vitro at days 4, 7, 14, 18 and 20 after the initiation of differentiation. Interestingly, although hiPSC demonstrated low levels of CD24 as has been previously reported [20], the intensity of CD24 expression gradually increased during chondrogenic differentiation with the hiChondrocytes consisting of a uniformly CD24^high^ population after differentiation and early cell passages (Fig. 1c). Upon comparing CD24 gene expression in five biological replicates of independently derived hiChondrocytes to adult chondrocytes, a 10-fold to 18-fold increase was consistently observed demonstrating that the hiChondrocytes were similar to the neonatal/juvenile chondrocytes in terms of CD24 expression (Fig. 1d). Collectively, these observations confirmed that CD24 is a cell surface receptor enriched in neonatal and juvenile chondrocytes as compared to adult chondrocytes.

### Differential inflammatory response in juvenile and adult chondrocytes

**Fig. 2:**
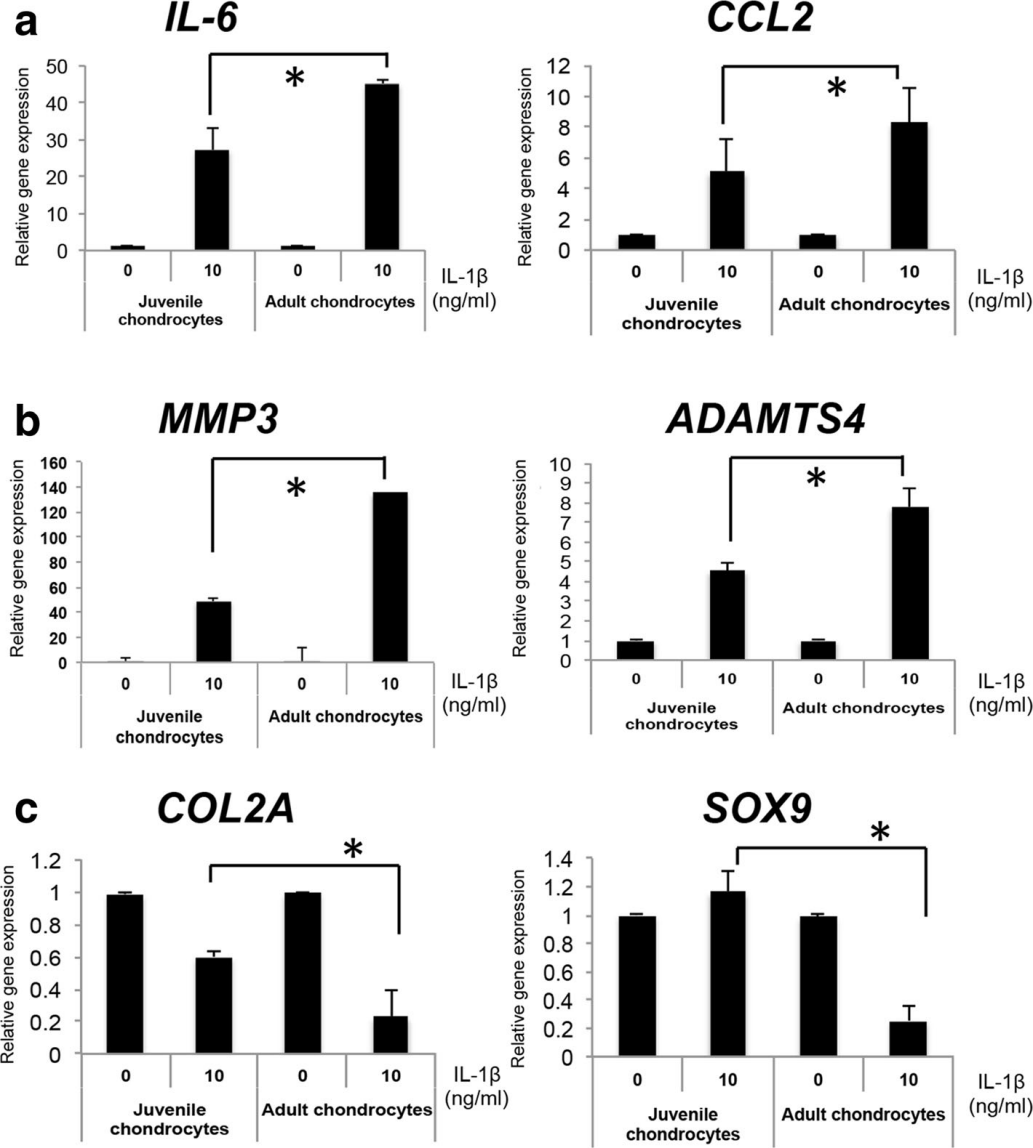
Differential inflammatory response in juvenile (*J1*, *J2*) and adult chondrocytes (*A1*, *A2*) upon IL-1ß stimulation (10 ng/mL) upon gene expression of inflammatory genes (*IL-6 and CCL2*) (**a**), catabolic genes (*MMP3 and ADAMTS4*) (**b**) and chondrocyte regulatory genes (*COL2A and SOX9*) (**c**); *p < 0.01. Gene expression is relative to control in the absence of IL-1ß for each respective gene

As CD24 has been shown to modulate innate immunity in immune cells [21], we questioned whether differential expression of CD24 in juvenile and adult chondrocytes will affect the response to pro-inflammatory cues. As IL-1ß plays a major role in inflammation in cartilage, we determined the response to IL-1ß treatment in CD24^high^ chondrocytes (juvenile chondrocytes) and CD24^low^ chondrocytes (adult chondrocytes). Upon IL-1ß treatment (0 or 10 ng/mL dose) of chondrocytes for 48 hours, we observed that the expression of inflammatory genes (*CCL2* and *IL-6*) was upregulated in both chondrocyte types - juvenile and adult chondrocytes (Fig. 2 and Figure S2, see Additional file 2). However, there was significantly greater upregulation of both *CCL2* and *IL-6* in the CD24^low^ adult chondrocytes as compared to the CD24^high^ juvenile chondrocytes (Fig. 2a and Additional file 2: Figure S2).

Similarly, upon testing a few catabolic genes, we observed significantly greater upregulation of *MMP3* and *ADAMTS4* in the CD24^low^ adult chondrocytes as compared to the juvenile CD24^high^ chondrocytes (Fig. 2b). In contrast, chondrogenic gene expression (*COL2A* and *SOX9*) was significantly decreased only in the CD24^low^ adult chondrocytes in response to IL-1ß treatment while the CD24^high^ juvenile chondrocytes were resistant and maintained the chondrogenic gene expression (Fig. 2c). Overall, the CD24^low^ chondrocytes had greater susceptibility to inflammatory cytokines.

### Loss of CD24 increases inflammatory response in chondrocytes

**Fig. 3:**
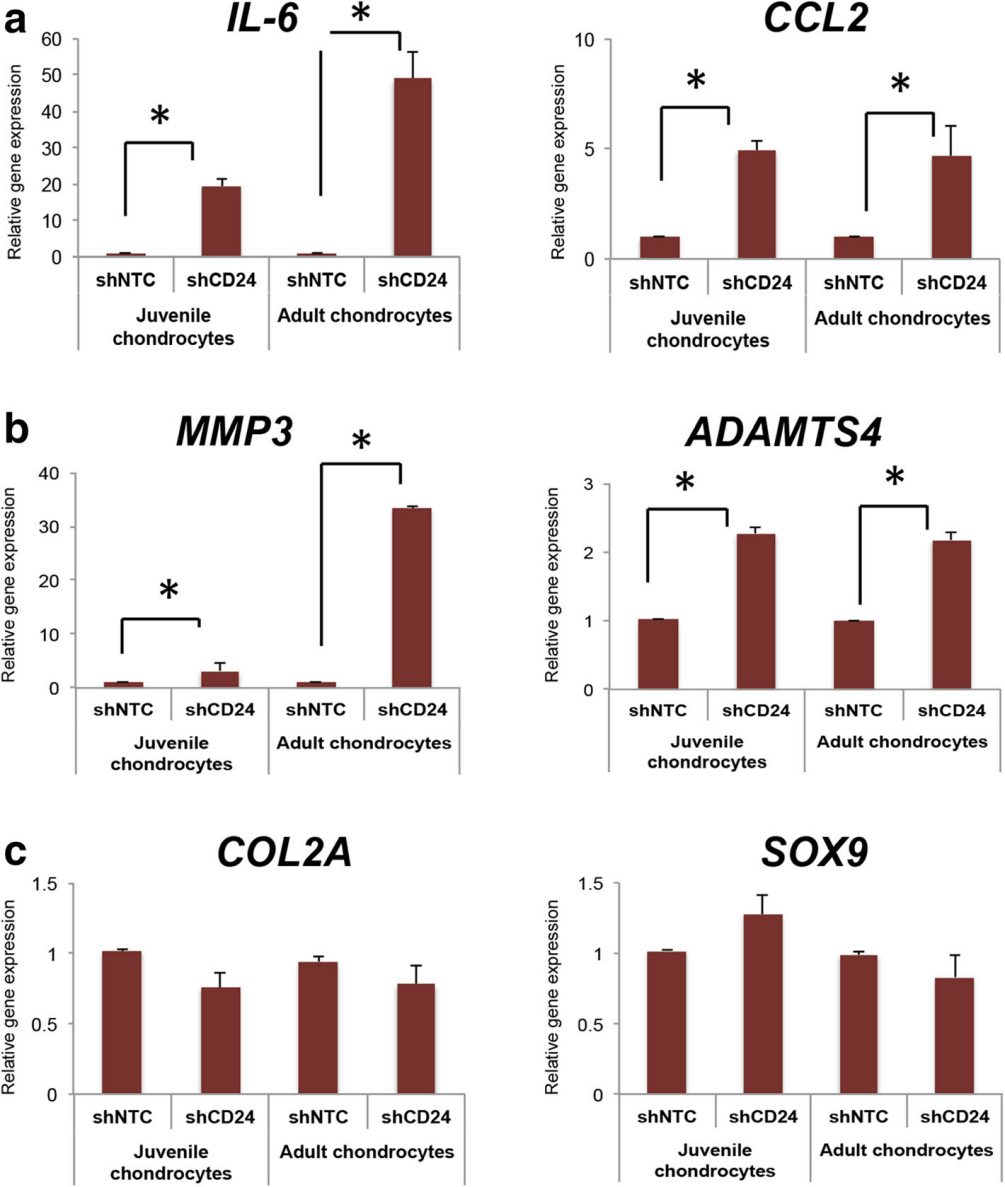
Loss of CD24 increases inflammatory gene expresson (**a**), and catabolic gene expression (**b**), but not chondrogenic gene expression (**c**), in both juvenile chondrocytes (*J1*, *J2*) and adult chondrocytes (*A1*, *A2*); *p < 0.01. Gene expression is relative to control short hairpin RNA (*shNTC*) for each respective gene

In order to further understand the effect of CD24 expression on the inflammatory and chondrogenic genes, we next investigated the effect of CD24 loss in the juvenile and adult chondrocytes. We firstly tested a set of five short hairpin (sh)RNAs and identified three independent shRNAs (sh1, 2 and 3; see “Methods”) that consistently showed an 80% or greater knockdown for CD24 at the gene and protein levels (Fig. 3 and Additional file 2). Real-time quantitative PCR was utilized to determine the mRNA levels and single-cell FACS analyses were used to confirm CD24 expression at the protein level. A non-target control shRNA was used along with the CD24 specific shRNA. Upon causing shRNA-mediated loss of CD24 in chondrocytes, we observed upregulation of the inflammatory genes *IL-6* and *CCL2* in juvenile and adult chondrocytes even in the absence of any stimulation with any pro-inflammatory cytokines (Fig. 3a). A similar increase was observed in the expression of catabolic genes, *MMP3* and *ADAMTS4*, upon loss of CD24; however, this increase was modest across the different chondrocytes (Fig. 3b). In contrast, loss of CD24 alone (that happens during normal aging) does not alter expression of the chondrogenic genes, *COL2A* and *SOX9* (Fig. 3c).

**Fig. 4:**
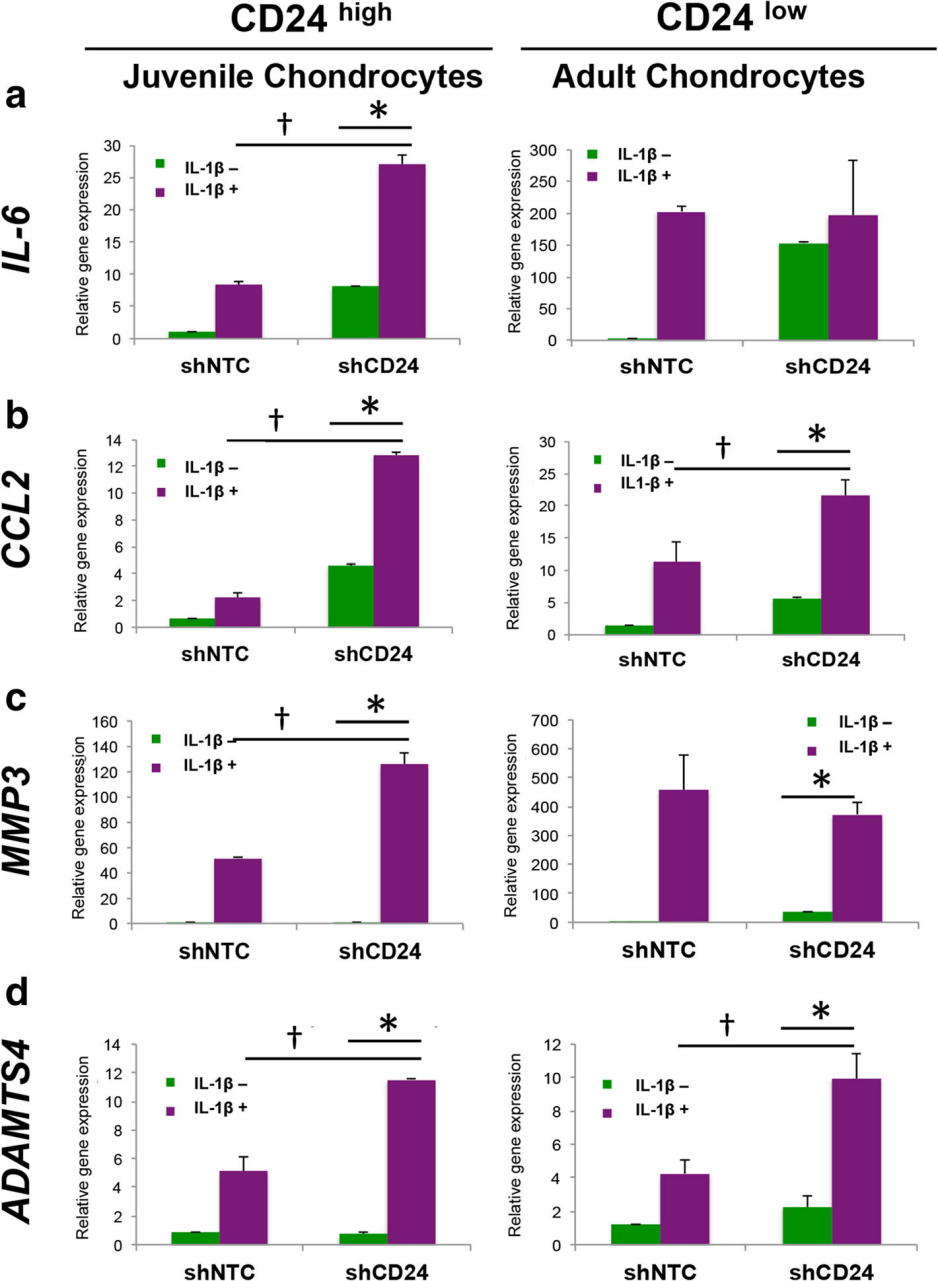
Loss of CD24 augments inflammatory response in the presence of IL-1ß (10 ng/mL). Gene expression for IL6 (**a**), CCL2 (**b**), MMP3 (**c**) and ADAMTS4 (**d**) in juvenile and adult chondrocytes upon control and shCD24 transduction in the absence and presence of IL-1ß; *p < 0.01 and †*p* < 0.01). *shNTC* control short hairpin RNA

Next, we assessed the effect of loss of CD24 in chondrocytes upon exposure to pro-inflammatory cytokines. Chondrocytes transduced with either the non-target control or CD24-specific shRNA were treated with IL-1ß treatment (0 or 10 ng/mL dose) for 48 hours. Gene expression of inflammatory (*IL-6* and *CCL2*), catabolic (*MMP3* and *ADAMTS4*) and chondrogenic (*COL2A* and *SOX9*) genes were then assayed using quantitative PCR. Loss of CD24 in combination with IL-1ß in chondrocytes enhanced inflammatory and catabolic gene expression, with significantly higher upregulation of *IL-6, CCL2, MMP3* and *ADAMTS4* in juvenile chondrocytes (Fig. 4). For adult chondrocytes that already had a small subset of cells expressing CD24, loss of CD24 further increased upregulation of *CCL2* and *ADAMTS4* significantly but not of *IL6* or *MMP3* in the presence of IL-1ß (Fig. 4). Interestingly, loss of CD24 rendered the juvenile chondrocytes susceptible to dedifferentiation in the presence of IL-1ß. In the presence of CD24 however, these chondrocytes were protected from the IL-1ß- mediated loss of *COL2A* and *SOX9* (see Additional file 2: Figure S4).

### CD24 inhibits NFκB activation in chondrocytes

**Fig. 5:**
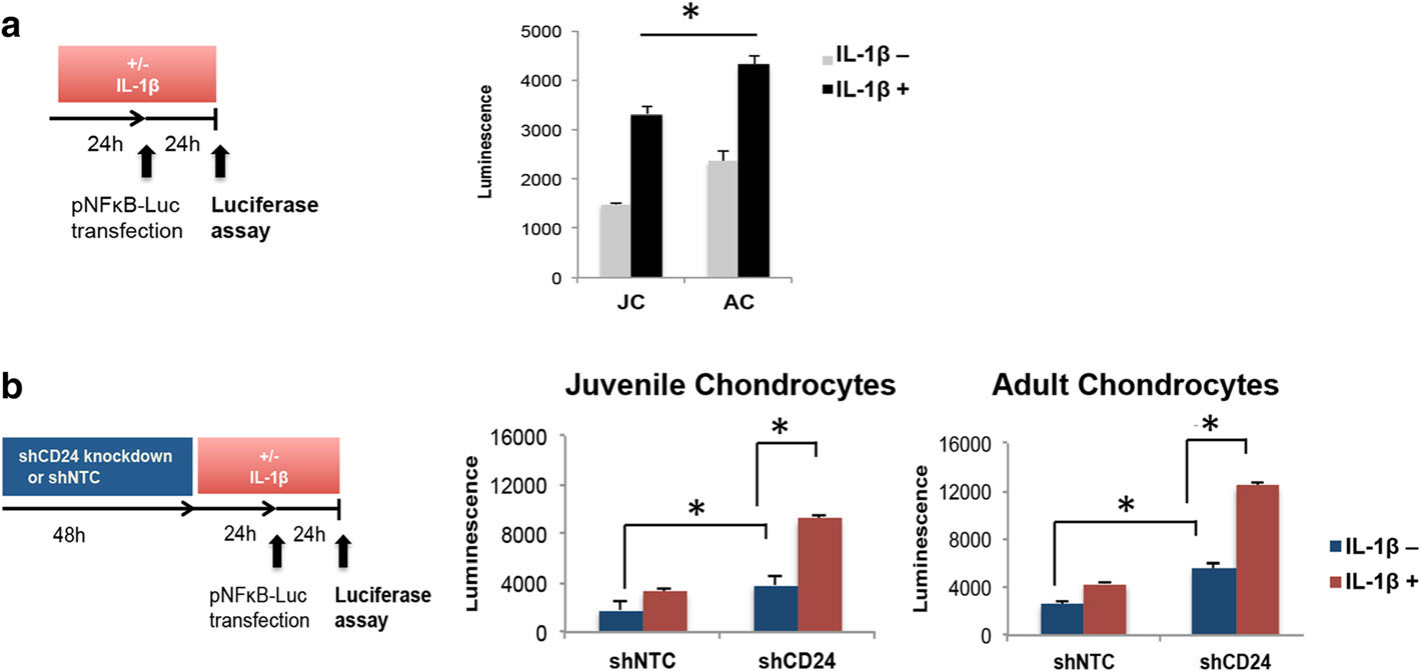
CD24 regulates nuclear factor kappa-activated B cell (NFκB) activity. **a** Using an NFκB-responsive luciferase construct, higher relative luminescence denoting higher NFκB activity is observed in adult chondrocytes (*AC*) compared to juvenile chondrocytes (*JC*) in the absence and presence of IL-1ß. **b** Loss of CD24 increases NFκB activity in the absence and presence of IL-1ß; *p < 0.01. *shNTC* control short hairpin RNA

The NFκB pathway is known to regulate expression of inflammatory and catabolic genes in OA. Previous studies have shown that CD24 signaling can inhibit NFκB activation in the immune system [21], therefore, we tested whether CD24 expression modulates NFκB activity in chondrocytes as well. NFκB activity was examined using NFκB reporter luciferase assay in the absence and presence of CD24. Upon transfection of NFκB-responsive luciferase construct in chondrocytes, we observed significantly higher relative luminescence in adult chondrocytes compared to juvenile chondrocytes in the absence and presence of IL-1ß (10 ng/mL) (Fig. 5a). Next, chondrocytes transduced with control shRNA (shNTC) or CD24 shRNA, were treated with IL-1ß (0 or 10 ng/mL dose) for 48 hours. After 24 hours of treatment, the NFκB-responsive luciferase construct was transfected in the chondrocytes, and assayed after another 24 hours (Fig. 5b). Increased relative luminescence, representing NFκB activity, was observed upon loss of CD24, which increased significantly upon IL-1ß treatment in all chondrocytes (Fig. 5b).

## Discussion

Juvenile chondrocytes (from donors under 13 years of age) have emerged in recent years as an attractive cell source for cartilage regeneration and tissue engineering. Differences between the juvenile and adult chondrocytes include increased proliferation and ECM generation in juvenile chondrocytes [22]. In addition, transplantation of allogeneic juvenile chondrocytes without any adverse effects and their inability to stimulate immune cells has suggests they are immune-privileged [1, 23]. In the present studies, we have additionally characterized hiPSC-derived chondrocytes (hiChondrocytes) that are a model for embryonic neonatal chondrocytes.

**Fig. 6:**
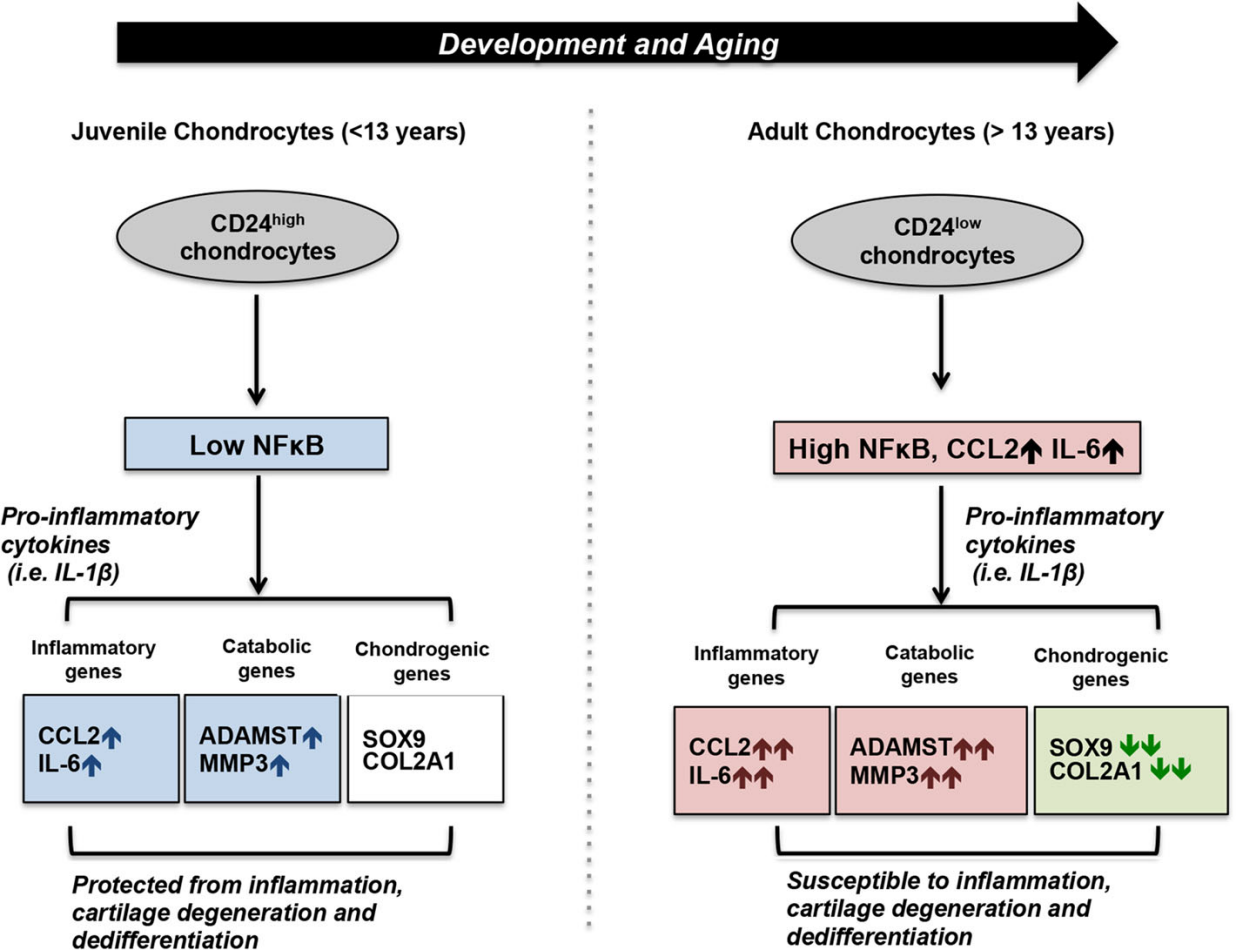
Schematic overview of the chondroprotective role of CD24 in cartilage

In studying hiChondrocytes (neonatal), juvenile chondrocytes (<13 years old donors) and adult chondrocytes (from donors aged 18–35 years), we are able to study a spectrum of early human cartilage development and aging, especially the response of these chondrocyte subsets to inflammatory cues. Interestingly, our studies demonstrate that juvenile chondrocytes and adult chondrocytes have a differential response to pro-inflammatory cues exemplified by IL-1ß. Upon IL-1ß stimulation, there was a greater upregulation of both inflammatory genes (such as *CCL2* and *IL-6*) and catabolic genes (*MMP3* and *ADAMTS4*) in adult chondrocytes compared to juvenile chondrocytes, revealing that the adult chondrocytes are more susceptible to inflammatory cues. In addition, the adult chondrocytes are more prone to dedifferentiation than the younger chondrocytes, as they had rapid loss of expression of chondrogenic genes, *COL2A* and *SOX9*, in the presence of IL-1ß. These results indicate that the juvenile chondrocytes are protected against inflammation and dedifferentiation and that this protection is gradually lost with aging (Fig. 6). Therefore, our studies highlight the fact that CD24^high^ juvenile articular chondrocytes with low immune responsiveness may have a distinctive advantage for cartilage repair especially in the highly inflammatory end-stage environment in RA and OA. In further studies, we would like to extend this characterization to chondrocytes from donors aged 40–60 years to discern whether the responsiveness to inflammatory cytokines is increased further with aging.

A central revelation of our studies is that CD24 is a novel molecular factor enriched in juvenile chondrocytes and regulates response towards inflammatory cues. Importantly, clinical studies have reported that the polymorphisms of human CD24 are associated with risk and progression of several autoimmune diseases, multiple sclerosis and rheumatoid arthritis (RA) [14–17]. In addition, CD24 expression and its prognostic significance have been reported for many types of cancer including breast, colorectal, gastric, lung, ovarian and pancreatic cancers, supporting CD24 as a diagnostic marker of cancer [24–27]. Although the clinical significance and function of CD24 in various diseases have been frequently reported, the regulatory and signaling mechanisms of CD24 are only beginning to be understood. CD24 does not contain a cytosolic domain, hence it needs to associate with and signal through another cell-surface receptor. In innate immune cells, CD24 has been shown to associate with Siglec-G, a member of the sialic acid-binding immunoglobulin-like lectin family in response to endogenous damage signals like high mobility group box 1 (HMGB1), heat shock protein 70 (HSP70) and heat shock protein 90 (HSP90) that are a part of the damage-associated molecular patterns (DAMP). Siglec-G contains cytosolic domains that inhibit NFκB, such that a loss of CD24 or Siglec-G can aberrantly activate NFκB. Both CD24-deficient and Siglec-G deficient mice, although viable, have been reported to demonstrate an intense response to induced inflammation in the liver, leading to acute and lethal liver damage [21, 28]. Blocking CD24 through soluble CD24, consisting of the extracellular portion of murine CD24 and human IgG1 Fc ameliorated the clinical symptom of experimental autoimmune disease, the mouse model of multiple sclerosis [29].

In the present studies, we demonstrate that the presence of high CD24 in the CD24^high^ juvenile chondrocyte populations keeps NFκB activation in check while a higher NFκB activity is observed in CD24^low^ adult chondrocytes. Silencing experiments that downregulated CD24 expression validated that CD24 is a negative modulator of NFκB activity. The higher NFκB activity in adult chondrocytes is accompanied by higher baseline expression of *CCL2* and *IL-6*, and upon cytokine stimulation it leads to augmented upregulation of inflammatory and catabolic genes along with rapid downregulation of chondrogenic genes (Fig. 6). Therefore, we have identified CD24 to be a novel modulator of the downstream NFκB pathway in chondrocytes. While the function of CD24 in immune cells has been in focus in autoimmune diseases and RA, our studies provide the important insight that changes in CD24 expression in cartilage additionally modulate the cartilage response towards inflammation in cartilage degenerative diseases. In future studies, we will explore the cartilage-specific CD24 cofactors (the Siglec family members for example) that interact with the components of the NFκB pathway and the mechanisms that ultimately lead to NFκB inhibition.

## Conclusions

Our studies provide new insights into the molecular mechanisms that underlie cartilage response to inflammatory cues during development, aging and disease. Decreased CD24 expression in chondrocytes during development and aging modulates NFκB activation and the intrinsic response of the chondrocytes to environmental inflammation. Inhibition of inflammatory modulators such as CD24 can also potentially constitute novel therapeutic strategies for cartilage degenerative diseases.

## Additional files

Additional file 1: Supplemental methods. (DOCX 73 kb)
Additional file 2:
**﻿﻿Figure S1.﻿﻿ **Immunofluorescence staining for CD24 in two independent adult cartilage tissues, *red* CD24-PE and *blue* DAPI (*scale bar* 100 um). **Figure S2.** Differential inflammatory response demonstrated in individual juvenile and adult chondrocytes (*J1*, *J2*, *A1*, *A2*) upon IL-1ß stimulation (10 ng/mL) to show the reproducibility of response. Gene expression of inflammatory genes (IL6 and CCL2) (A) and catabolic genes (MMP3 and ADAMTS4) and chondrocyte regulatory genes (COL2A and SOX9) (B) in the absence and presence of IL-1ß. Gene expression is relative to J1 in the absence of IL-1ß for each respective gene. **Figure S3.** A CD24 gene expression upon transduction of shRNAs against CD24 (*sh1*–*sh5*) in chondrocytes. B CD24 expression upon shCD24 knockdown (*sh1*, *sh2*) in juvenile (*J1*, *J2*) and adult chondrocytes (*A1*, *A2*). C Flow cytometry analyses confirmed reduction in CD24-positive cells in juvenile and adult chondrocyte populations upon shCD24 transduction. **Figure S4.** Loss of CD24 synergistically enhances downregulation of chondrogenic genes (A) Sox9 and (B) Col2a1, in the presence of IL-1ß (10 ng/mL) in juvenile and adult chondrocytes; **p* < 0.01. (PDF 757 kb)

## Abbreviations

AC: adult articular chondrocytes
BSA: bovine serum albumin
CD24: cluster of differentiation 24
ECM: extracellular matrix
FACS: fluorescence-activated cell sorting
GAPDH: glyceraldehyde-3-phosphate dehydrogenase
hiChondrocytes: human induced pluripotent stem cell-derived chondrocytes
hiPSC: human induced pluripotent stem cells
IL-1β: interleukin-1 beta
IL-6: interleukin-6
JC: juvenile articular chondrocytes
NFκB: nuclear factor kappa-activated B cells
OA: osteoarthritis
PBS: phosphate-buffered saline
RA: rheumatoid arthritis
shRNA: short hairpin RNA
Sox9: sex-determining region Y (SRY)-box 9
