# Supplementary material for: CD24 enrichment protects while its loss increases susceptibility of juvenile chondrocytes towards inflammation
Source: Arthritis Res Ther. 2016 Dec 12;18:292. doi: 10.1186/s13075-016-1183-y (PMC5153697; doi:10.1186/s13075-016-1183-y)
Supplement: Additional file 1: — Supplemental methods. (DOCX 73 kb) [file 13075_2016_1183_MOESM1_ESM.docx]

**Supplemental methods**

**Statistical analyses**

One experimental gene expression for all the samples was relative to one reading of the sample A1 out of three independent experiments such that we are able to obtain the variation (standard deviation) for the sample A1 as well as A2-A4, J1-J4 and hiChondrocytes (1-5) (Figure 1A/1D). A t-test was then utilized to discern whether the juvenile samples (J1-J4) are different from the adult samples (A1-A4) in a statistically significant way. For datasets showing fold changes to controls, we have similarly utilized the experimental gene expression for one sample say A1 out of three independent experiments to quantitate relative expression for all the other samples, such that we are able to obtain the variation (standard deviation) for the sample A1 as well as A2-A4 in the absence and presence of IL1β (Figures 2, 3). For the data in Figure 4, a one-way ANOVA was utilized followed by the Bonferroni’s test for multiple-comparisons. P-values less than 0.01 were considered significant.
