# Supplementary material for: CD24 enrichment protects while its loss increases susceptibility of juvenile chondrocytes towards inflammation
Source: Arthritis Res Ther. 2016 Dec 12;18:292. doi: 10.1186/s13075-016-1183-y (PMC5153697; doi:10.1186/s13075-016-1183-y)
Supplement: Additional file 2: — ﻿﻿Figure S1.﻿﻿ Immunofluorescence staining for CD24 in two independent adult cartilage tissues, red CD24-PE and blue DAPI (scale bar 100 um). Figure S2. Differential inflammatory response demonstrated in individual juvenile and adult chondrocytes (J1, J2, A1, A2) upon IL-1ß stimulation (10 ng/mL) to show the reproducibility of response. Gene expression of inflammatory genes (IL6 and CCL2) (A) and catabolic genes (MMP3 and ADAMTS4) and chondrocyte regulatory genes (COL2A and SOX9) (B) in the absence and presence of IL-1ß. Gene expression is relative to J1 in the absence of IL-1ß for each respective gene. Figure S3. A CD24 gene expression upon transduction of shRNAs against CD24 (sh1–sh5) in chondrocytes. B CD24 expression upon shCD24 knockdown (sh1, sh2) in juvenile (J1, J2) and adult chondrocytes (A1, A2). C Flow cytometry analyses confirmed reduction in CD24-positive cells in juvenile and adult chondrocyte populations upon shCD24 transduction. Figure S4. Loss of CD24 synergistically enhances downregulation of chondrogenic genes (A) Sox9 and (B) Col2a1, in the presence of IL-1ß (10 ng/mL) in juvenile and adult chondrocytes; *p < 0.01. (PDF 757 kb) [file 13075_2016_1183_MOESM2_ESM.pdf]

CD24 expression in adult cartilage(AC) tissue

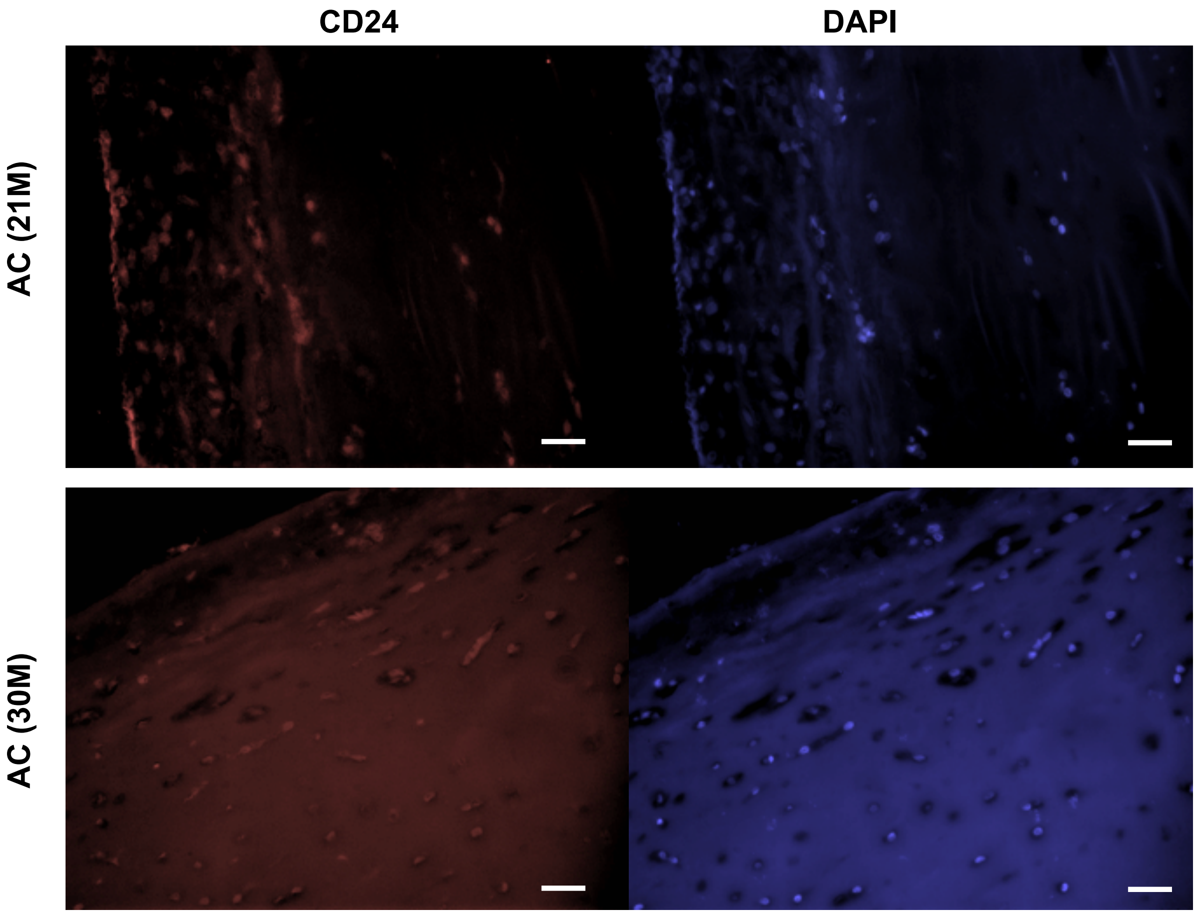

Fig. S1 (related to Fig. 1). Immunofluorescence staining for CD24 in two independent adult cartilage tissues, Red: CD24-PE and Blue: DAPI (Scale bar 100um).

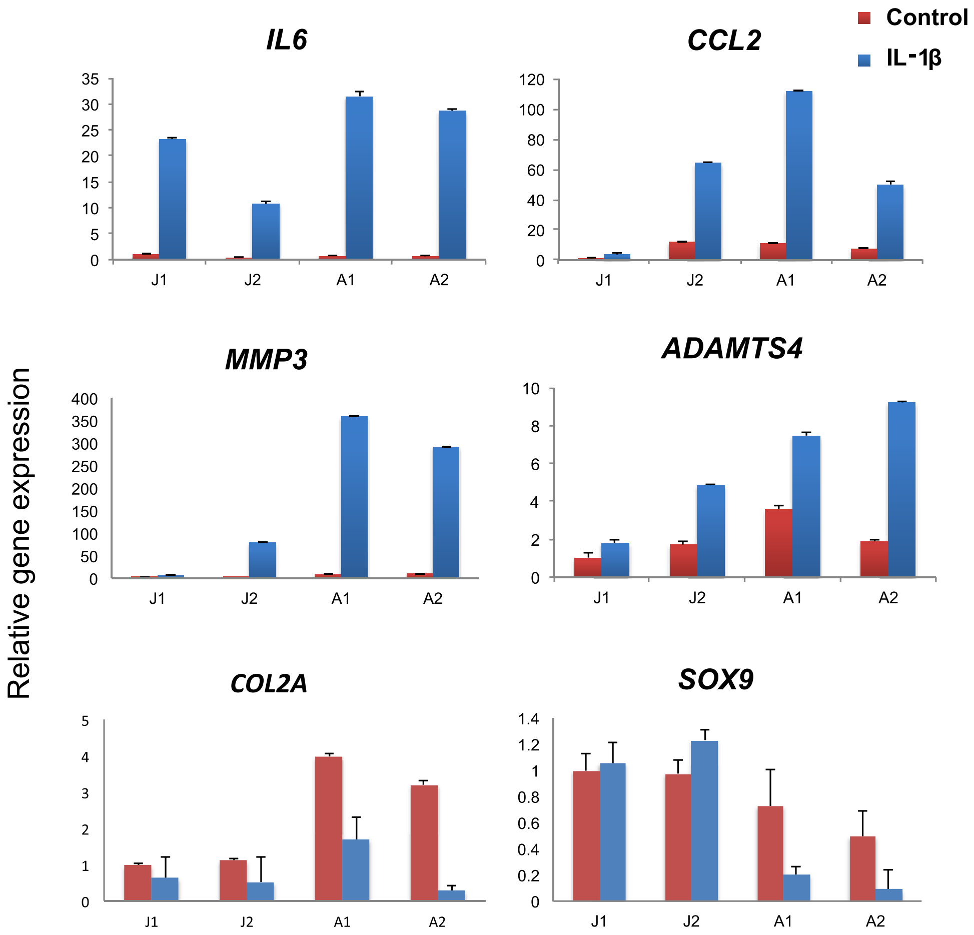

Fig. S2 (related to Fig. 2). Differential inflammatory response demonstrated in individual juvenile and adult chondrocytes (J1, J2, A1, A2) upon IL1ß stimulation (10ng/ml) to show the reproducibility of response. Gene expression of (A) inflammatory genes *(IL6 and CCL2*), (B) catabolic genes (*MMP3 and ADAMTS4*) and chondrocyte regulatory genes (*COL2A and SOX9*) in the absence and presence of IL1ß. Gene expression is relative to J1 in the absence of IL1ß for each respective gene.

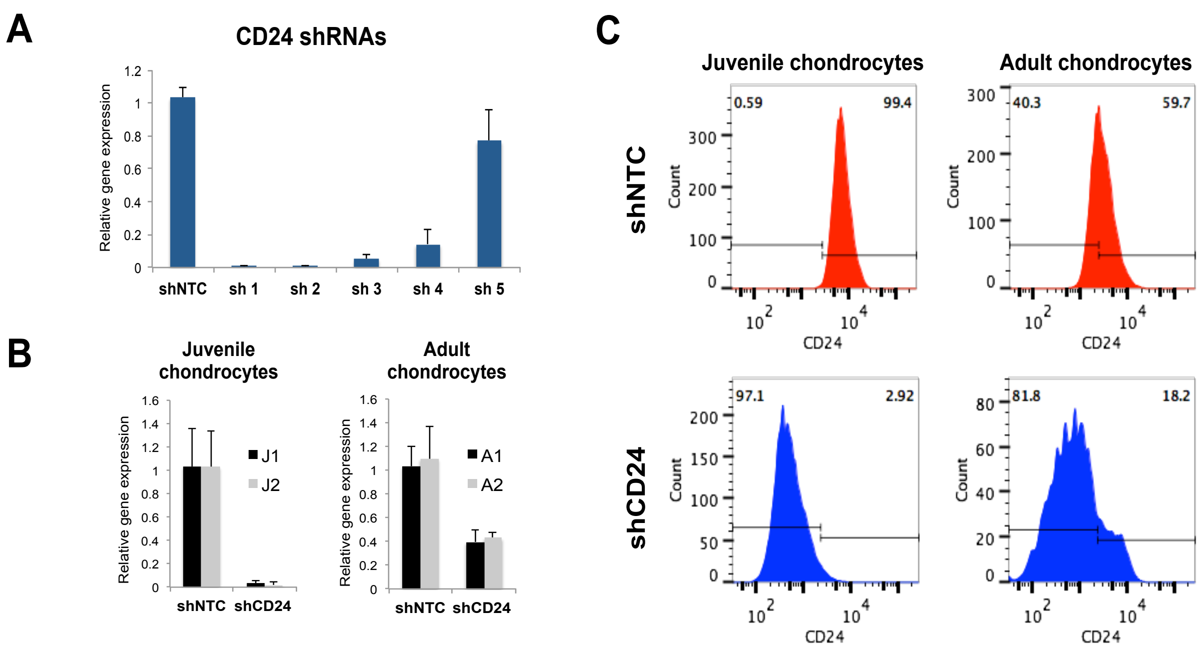

Fig. S3. (related to Fig. 3). (A) CD24 gene expression upon transduction of shRNAs against CD24 (sh1-sh5) in chondrocytes, (B) CD24 expression upon shCD24 knockdown (sh1, sh2) in juvenile (J1, J2) and adult chondrocytes (A1, A2), (C) Flow Cytometry analyses confirmed reduction in CD24 positive cells in juvenile and adult chondrocyte populations upon shCD24 transduction.

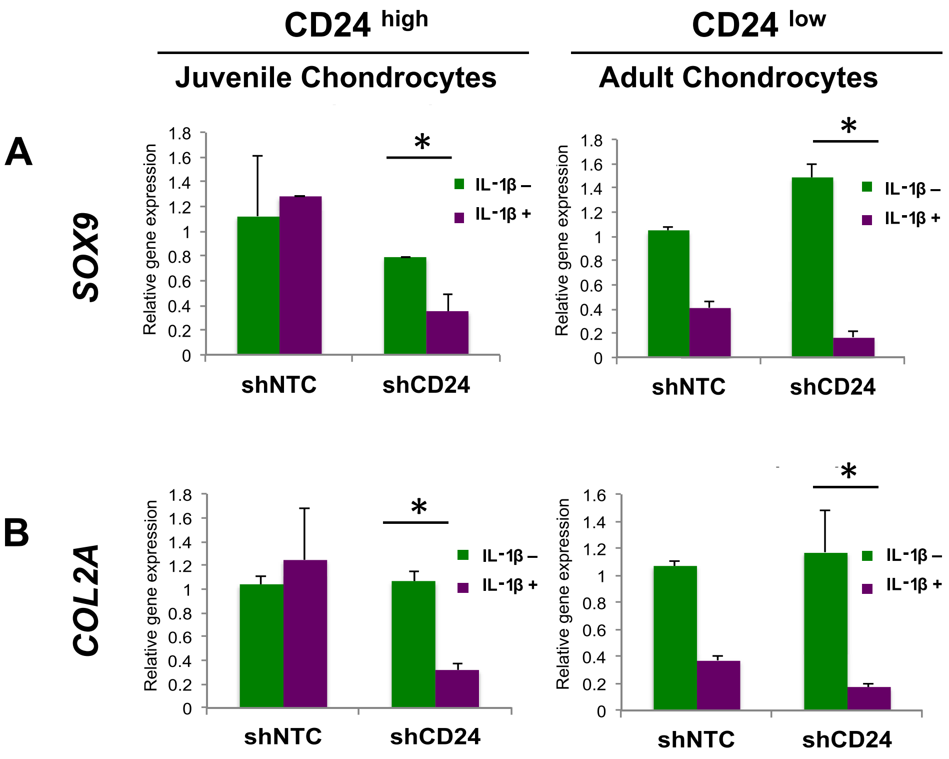

Fig. S4. (related to Fig. 4). Loss of CD24 synergistically enhances downregulation of chondrogenic genes (A) Sox9 and (B) Col2a1, in the presence of IL1ß (10ng/ml) in juvenile and adult chondrocytes, (* denotes $p<0.01$).
